# Removal of Methylene Blue from Aqueous Solutions by Surface Modified Talc

**DOI:** 10.3390/ma16093597

**Published:** 2023-05-08

**Authors:** Shuyang Chen, Mei Zhang, Hanjie Chen, Ying Fang

**Affiliations:** College of Materials Science and Engineering, Nanjing Tech University, Nanjing 211816, China

**Keywords:** modified talc, adsorption process, methylene blue, isotherms, kinetics

## Abstract

In this study, raw talc powder surface modification was conducted, and the powder was modified in two different methods using acid washing and ball milling. Modified talc was characterized by X-ray diffraction (XRD), Fourier transforms infrared spectroscopy (FTIR), and scanning electron microscopy (SEM). In order to investigate the adsorption capacity of modified talc on dyes, adsorption experiments were carried out with methylene blue (MB) in aqueous solutions as the target contaminant. The findings of the characterization revealed that both modifications increased the adsorption capacity of talc, which was attributed to changes in specific surface area and active groups. The influence of process parameters such as contact time, pH, dye concentration, and adsorbent dosage on the adsorption performance was systematically investigated. Modified talc was able to adsorb MB rapidly, reaching equilibrium within 60 min. Additionally, the adsorption performance was improved as the pH of the dye solution increased. The isotherms for MB adsorption by modified talc fitted well with the Langmuir model. The pseudo-second-order model in the adsorption kinetic model properly described the adsorption behavior. The results show that the modified talc can be used as an inexpensive and abundant candidate material for the adsorption of dyes in industrial wastewater.

## 1. Introduction

Dye wastewater contains organic substances such as azo bonds and polyaromatic rings due to its deep chromaticity and complex molecular components, making it stable and less susceptible to biodegradation [1]. Dyes in dye wastewater reduce light transmission and impede plant growth, as well as their chelated metal ions are toxic to both aquatic animals and microorganisms [2,3,4]. Some dyes are non-degradable organic substances and their mutagenic, teratogenic, and carcinogenic properties pose a significant risk to human health [5,6,7,8]. As a result, dye wastewater is highly hazardous and harmful, and it has affected the development of paper, plastics, textiles, pharmaceuticals, and food [9,10,11]. Moreover, dye wastewater production will keep growing in the coming years. Currently, a wide range of methods have been investigated for the removal of dye wastewater, including chemical oxidation, membrane separation, semiconductor photocatalysis, and adsorption [12,13,14,15]. Among them, adsorption is an effective method for removing dyes from wastewater due to its simple operation and low cost. One of the most efficient adsorbents for wastewater treatment is activated carbon, which has a large surface area, strong adsorption capacity, is porous, and has a long history of use. According to studies [16], carboxyl modification of activated carbon surfaces can enhance adsorption performance. However, the high costs of manufacturing and regenerating activated carbon have limited its large-scale application in adsorption [17].

Compared to natural materials and metal oxides, natural minerals have become a research hotspot for wastewater adsorption due to their abundance, large specific surface area, special structure, and exchangeable cations [18,19,20,21,22,23]. Talc is a lamellar silicate mineral containing MgO and is chemically stable, Si can be replaced by Al and Mg can be replaced by Fe, Mn, Ni, and Al. Talc has a trioctahedral structure and is a monoclinic crystal system. Most of the crystals are flaky dense blocks, and few are plate-like, with perfect cleavage between layers [24,25]. The charge within the structural unit layer is balanced and the interlayer molecular bond and van der Waals forces are weak [26]. Talc is an environmentally friendly and efficient adsorbent. The ultrafine powder can be used to treat colored components of dyes and pigments [27,28]. In addition, talc also has a good adsorption effect on heavy metal ions such as Pb^2+^, Ni^2+^, and Cu^2+^ due to its active functional groups, unique pore structure, and chemical stability [29,30,31,32]. In recent years, natural clay adsorbents have received more and more attention and research in wastewater treatment. With excellent adsorption results, clay minerals prepared using a variety of modification techniques have been used successfully for wastewater treatment [33,34,35,36,37]. Li et al. [38] modified talc by microwave-assisted acidification. The heat generated by the microwave entered the talc to destroy its internal structure, the water, and impurities were removed from the talc under the action of radiation and acid, and the pores of the talc increased, greatly increasing the removal efficiency of contaminants. Due to the large amount of impurities and carbonates found in natural talc minerals, the adsorption of contaminants is limited and the regeneration is difficult. Therefore, surface modification of talc is necessary to improve its adsorption performance.

In this study, we reported two simple talc modification techniques by acid washing modification and ball milling modification, and adsorption experiments were conducted with the typical cationic dye methylene blue (MB) as the target contaminant to investigate the removal capacity of modified talc on dye wastewater. The effect of different modification methods on the physicochemical properties of talc was explored through characterization. The effects of adsorption time, pH, dye concentration, and adsorbent dosage on the adsorption process were examined. In addition, adsorption kinetics and adsorption isotherms were discussed to investigate the adsorption mechanism. The innovation of this paper is that the ball milling method optimizes the process by combining mechanical force effects with surface chemical modification. The acid washing method erodes the surface of the talc, resulting in a more complete surface modification. These two modification methods are simple in process, low in economic cost green and pollution-free, and have a good modification effect.

## 2. Materials and Methods

### 2.1. Materials

**Figure 1:**
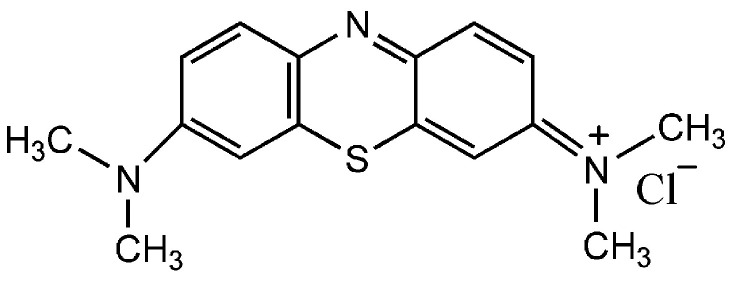
The molecular structure of MB.

Raw material 800 mesh talc (Mg_3_Si_4_O_10_(OH)_2_) from Shanghai Macklin Biochemical Co., Ltd. Nitric acid (HNO_3_, 68%) supplied by Shanghai Lingfeng Chemical Reagent Co., Ltd., Shanghai, China. Cetyltrimethylammonium bromide (CTAB, 99%) and methylene blue (MB, 98.5%, C.I.: 52015) were obtained from Sinopharm Chemical Reagent Co., Ltd., Shanghai, China. The molecular structure of MB is shown in Figure 1. All chemicals were of analytical reagent grade and used without further purification. The water used in the experiments was deionized water at room temperature.

### 2.2. Surface Modifications of Talc

The ball milling modification started by drying the raw talc at 110 °C to a constant weight. Agate balls and 15 g talc were then added to the ball mill jar, along with a certain amount of modifier CTAB (1%) relative to the sample, and ground with a planetary high energy ball mill (Pulverisette 4, Fritsch, Oberstein, Germany). The ball milling speed was 250 rpm, the ball-to-powder mass ratio was 5:1, the number of small and large balls (8 and 10 mm diameter) was 3:1, and the ball milling time was 3 h. The modified sample was washed with deionized water to remove unreacted CTAB until no Br^−^ was detected (0.01 mol/L AgNO_3_), dried at 110 °C, and then sieved to obtain M-Talc.

Acid washing modification was carried out by stirring 25 g of raw talc with deionized water in a ratio of 1:10 at room temperature for 2 h, vacuum filtering, washing several times, drying at 110 °C, and then grinding. Talc powder was added to 250 mL of 30% volume fraction of nitric acid solution and stirred continuously at 40 °C for 3 h. The modified talc was filtered and washed until the aqueous solution was neutral and then dried at 110 °C. Next, it was calcined under vacuum at 200 °C for 2 h, ground, and sieved after cooling to obtain A-Talc.

### 2.3. Characterization

The samples structures were characterized using an X-ray diffractometer (XRD) (D2 Phaser, Bruker, Germany) with Cu Kα radiation in the 2θ range of 5−80° with a rated power of 3 kW and a scanning speed of 10°/min. The changes in the functional groups on the adsorbent surface were detected using Fourier transform infrared spectroscopy (FTIR) (Nicolet iS20, Thermo Scientific, Waltham, MA, USA) in the wavenumber range of 400−4000 cm^−1^. The surface morphology was observed using a scanning electron microscope (SEM) (Gemini 300, ZEISS, Jena, Germany) at an accelerating voltage of 3 kV. Particle size and specific surface area were measured using a laser particle size analyzer (Mastersizer 2000, Malvern, PA, USA).

### 2.4. Adsorption Experiments

In this study, an MB solution was used to simulate industrial dye wastewater to determine the adsorption effects of modified talc. To prepare the solution, 0.1 g of MB dye was dissolved in 1 L of water, and the required concentration was obtained by further dilution. A certain amount of adsorbent was weighed into a 250 mL beaker, and 100 mL of the MB solution was added. The mixture was stirred magnetically for a given time and then left to stand. After adsorption, the supernatant was separated by centrifugation at 4000 rpm for 10 min, and the residual MB dye concentration was measured by a 721 UV-vis spectrophotometer at 666 nm. The effects of various factors on the adsorption process, such as adsorption time (0–120 min), adsorbent dosage (0.2–1 g), pH (2–11), and initial dye concentration (5–15 mg/L), were investigated at a constant temperature of 30 °C. The removal efficiency *R* (%) and the adsorption capacity *q_e_* (mg/g) for MB were calculated using the following equations:

(1) $$R=\frac{C_{0}-C_{e}}{C_{0}}\times 100\%$$

(2) $$q_{e}=\frac{\left(C_{0}-C_{e}\right)\times V}{m}$$

where *C*_0_ (mg/L) is the initial concentration of the dye, *C_e_* (mg/L) is the equilibrium concentration of the dye, *m* (g) is the mass of the adsorbent and *V* (L) is the volume of the dye solution.

## 3. Results and Discussion

### 3.1. Characterization of Raw Talc and Modified Talc

**Figure 2:**
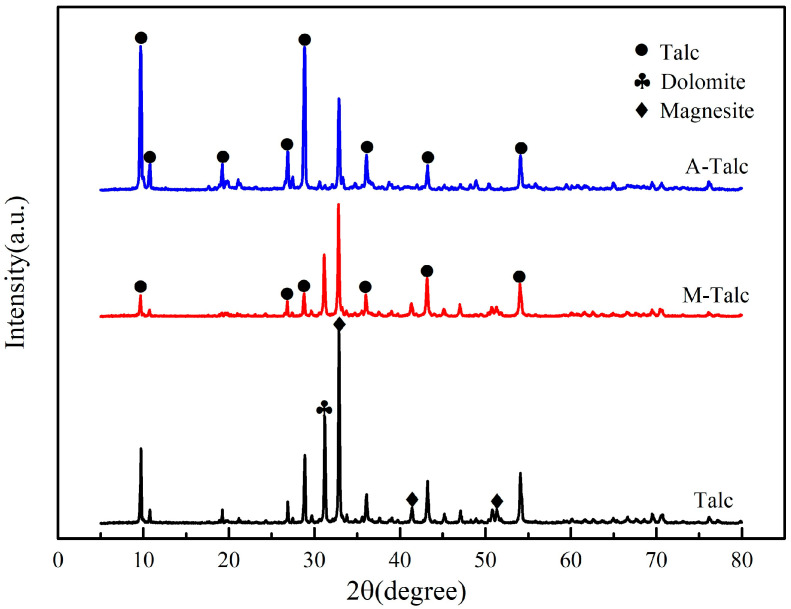
XRD patterns of raw talc, M-Talc, and A-Talc.

Figure 2 demonstrates the XRD spectra of raw talc and modified talc. Raw talc contained magnesite (2θ = 32.89, 41.41, 51.35°) and dolomite (2θ = 31.21°) impurities and the magnesite diffraction peaks were strong, indicating that the talc contained more magnesite impurities. After ball milling modification, the characteristic diffraction peaks of M-Talc (2θ = 9.71, 10.74, 28.88°) were weakened and some of the peaks disappeared, and the crystal structure of talc was disrupted to a certain extent and partially amorphization due to the weaker van der Waals force between the layers, which provides the possibility of enhanced particle activity in talc powder. In addition, the layer spacing of the talc did not change, suggesting that CTAB did not enter the interlayer of the talc, but only covered its surface. A-Talc peaks became sharper after acid modification, which may be related to crystal size and the mean lattice strain [39]. The characteristic diffraction peaks (2θ = 9.65, 19.25, 28.84°) correspond to the (002), (004), and (006) crystal planes, respectively, as a set of parallel cleavage planes. Furthermore, the diffraction peaks of the impurities dolomite and magnesite weaken or even disappeared completely, indicating that most of the carbonate impurities had been dissolved by the acid solution and the pores became dredged.

**Figure 3:**
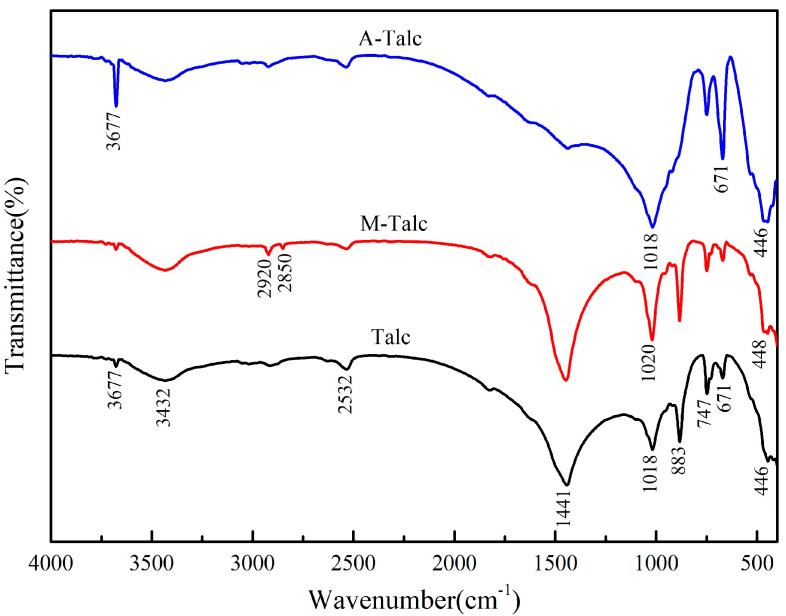
FTIR spectra of raw talc, M-Talc, and A-Talc.

The FTIR spectra of raw and modified talc are recorded in Figure 3. The FTIR spectra show the characteristic absorption peaks in raw talc: Mg-OH stretching vibration (3677 cm^−1^), O-H stretching vibration of crystal water (3432 cm^−1^), O-H bending vibration (671 cm^−1^), Si-O stretching and bending vibration (1018 cm^−1^ and 446 cm^−1^) [40,41]. Compared to Talc, M-Talc showed new absorption peaks at 2920 cm^−1^ and 2850 cm^−1^, which were stretching vibrations of -CH_3_ and -CH_2_ in CTAB, indicating successful loading of CTAB on the talc surface. Moreover, the absorption peaks of M-Talc at 1018 cm^−1^ and 446 cm^−1^ were slightly shifted towards the higher wavenumbers due to the strong mechanical forces acting to change the bond energy and bond structure of the Si-O bond, the internal energy of the system increases and the talc is activated. The increased intensity of the absorption peaks of Si-O at 1018 cm^−1^ and 446 cm^−1^ and the sharpness of the peaks of A-Talc indicates that more Si-O reactive groups appeared on the surface of the talc after acid activation, and the enhancement of the absorption peaks at 3677 cm^−1^ and 671 cm^−1^ was also evidence of the acid reaction. Furthermore, the absorption peaks of impurities dolomite (2532 cm^−1^ and 1441 cm^−1^) and magnesite (883 cm^−1^ and 741 cm^−1^) weaken or even disappeared, and the purity of talc became higher, consistent with the results of XRD analysis.

**Figure 4:**
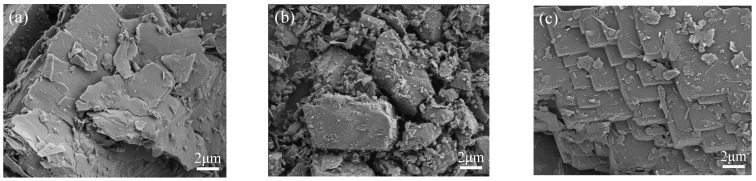
SEM images of raw talc (**a**), M-Talc (**b**), and A-Talc (**c**).

The surface morphology of the raw talc and modified talc are presented by SEM analysis. From Figure 4 it can be seen that the raw talc crystal form has a lamellar structure and lamellar lines. The particle size D_50_ is around 13.1 μm (Table 1), with multiple layers of lamellae of varying sizes, and the surface of the particles is relatively rough. At the end of three hours of milling, the M-Talc lamellar structure was broken. The particle size was reduced from 13.1 to 3.1 μm and the morphology of the talc was changed. Ball milling produced irregularly shaped and loosely arranged fine particles. In addition, small agglomerates appeared on the surface of the particles, as a result of the ball milling causing the larger particles to become smaller and then aggregate together. The CTAB loading on the talc surface acted as a modifier. This morphology and structure improved the adsorption performance of the talc. After acid and heat treatment, the typical lamellar structure of A-Talc crystals was evident and neat. Layers were stacked on top of each other, creating a larger lamellar structure than the raw talc. The specific surface area increased from 1.3 to 4.5 m^2^/g, allowing a large number of active sites to be exposed and facilitating the adsorption of contaminants.

### 3.2. Adsorption Capacities of Raw Talc and Modified Talc

**Figure 5:**
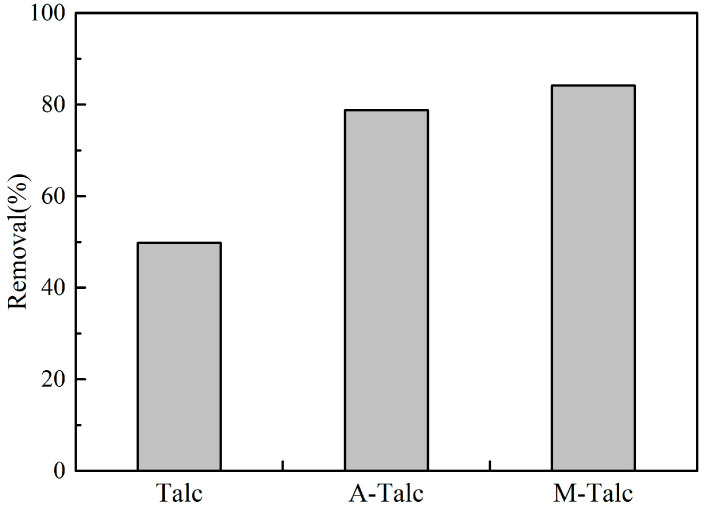
The removal of MB by talc and modified talc.

A total of 0.4 g of raw talc and modified talc were added to 100 mL of MB solution at a concentration of 5 mg/L at pH 7. The supernatant was centrifuged after 60 min of magnetic stirring to determine the adsorption performance of the specimens. Figure 5 shows a comparison of the adsorption performance on MB by three adsorbents of raw and modified talc. Under the same experimental conditions, the removal of MB by raw talc, A-Talc, and M-Talc was 49.81, 78.75, and 84.16% respectively. The enhancement of M-Talc removal may be due to the decrease in particle size and increase in specific surface area under the action of mechanical forces. On the other hand, CTAB loading on the talc surface changes the talc surface from a hydrophobic to a hydrophilic organic state. The improved adsorption performance of A-Talc on MB is due to the dissolution of impurities in the pore channels, the pore channels become sparse and the active functional groups increase. The results suggested that surface modification could significantly enhance the adsorption of contaminants by talc. The absorbents had better adsorption capacity for MB compared to other modified talc [42,43]. Therefore, two modified talc were selected for further experimental studies.

### 3.3. Effect of Contact Time on Adsorption

**Figure 6:**
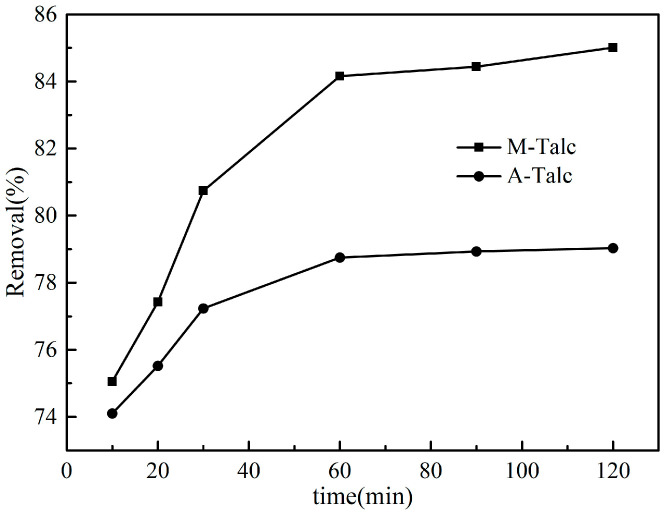
The effect of contact time on the removal of MB by M-Talc and A-Talc (*C*_0_ = 5 mg/L, dosage = 4 g/L, pH = 7).

Contact time is one of the most important factors affecting adsorption performance and fast adsorption rates are more favorable for practical applications. The adsorption lasted for 120 min and the optimum adsorption contact time was detected. Figure 6 shows the effect of different contact time-modified talc on MB removal. The results demonstrated that the removal of MB by M-Talc and A-Talc increased with increasing contact time in the early stage of adsorption. The adsorption capacity of M-Talc was better than that of A-Talc and both reached equilibrium at 60 min. Further extension of the time did not result in a significant increase in the removal rate. Therefore, the adsorption process could be considered in two stages: the first stage resulted in a fast adsorption rate due to the presence of a large number of unoccupied adsorption sites on the surface of the adsorbent. As the adsorption process continued, the adsorption sites were essentially saturated and then gradually reduced, resulting in a second stage that was a slow adsorption process that eventually reached equilibrium. Based on experimental data, the optimum contact time was 60 min.

### 3.4. Effect of pH on Adsorption

**Figure 7:**
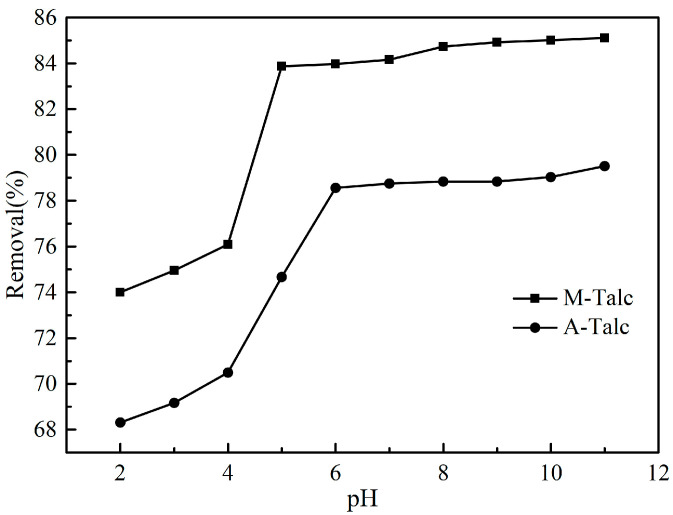
The effect of pH on the removal of MB by M-Talc and A-Talc (*C*_0_ = 5 mg/L, dosage = 4 g/L, *t* = 60 min).

As pH controls the ionization process between the adsorbent and the adsorbate, it is considered to be one of the most significant factors affecting dye adsorption. To investigate the effect of pH on MB adsorption, initial conditions were kept constant and the pH was adjusted from 2 to 11 using 0.1 mol/L HCl and 0.1 mol/L NaOH. The point of the zero charge (pH_pzc_) was determined using the previously reported method [44]. The removal of MB by the two modified talc at different pH conditions is illustrated in Figure 7. The results indicated that the removal of M-Talc and A-Talc increased with increasing pH when pH was less than 6, and the removal of MB was essentially constant from pH 6 to 11. This phenomenon can be explained by the change of adsorbent surface charge with pH. The pH_pzc_ of M-Talc and A-Talc was around 6.5 and 5.9 respectively. When the pH was less than pH_pzc_, the higher concentration of hydrogen ions competed with the cationic dye MB for available active sites, and there were repulsive forces between the positive charge on the talc surface and the MB, which hindered the adsorption process of the contaminant by the adsorbent, hence the relatively low removal of MB. On the other hand, as the pH increased, the surface of the modified talc was negatively charged and the electrostatic attraction was stronger, which facilitated the adsorption of MB [45]. The above studies showed that both modified talc were effective in removing MB under neutral or alkaline conditions.

### 3.5. Effect of Initial Dye Concentration on Adsorption

**Figure 8:**
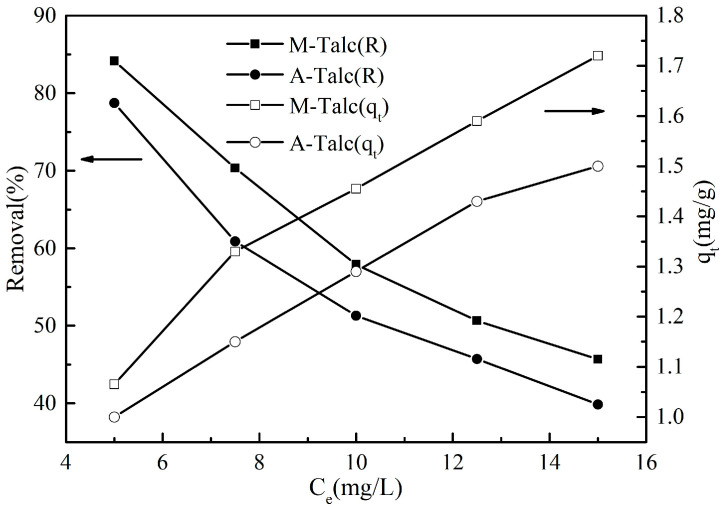
The effect of the initial concentration of MB on the adsorption by M-Talc and A-Talc (dosage = 4 g/L, *t* = 60 min, pH = 7).

The initial concentration of the dye provides the driving force to overcome the mass transfer resistance of the molecule between the solid and the liquid phase, so the adsorption capacity of the adsorbent to MB is related to the concentration. Different concentrations of MB were selected and other conditions were kept unchanged to investigate the effect of the initial dye concentration on the adsorption performance of the modified talc. As seen in Figure 8 the removal of MB by M-Talc and A-Talc showed a similar downward trend as the concentration of MB increased. Conversely, the adsorption capacity increased with the increase in MB concentration. This is because, at higher MB concentrations, more dye cations appear on each active site on the adsorbent surface and the active sites are saturated to a certain extent, leading to a decrease in removal [46]. Likewise, the adsorbent surface became more in contact with the dye, thus increasing the adsorption rate and adsorption capacity. From the above analysis, it was clear that the two modified talc were more effective in removing MB at low concentrations.

### 3.6. Effect of Adsorbent Dosage on Adsorption

**Figure 9:**
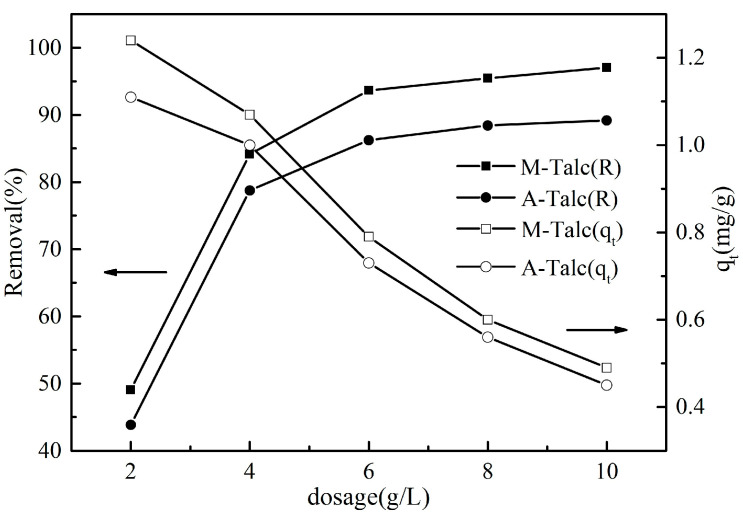
The effect of adsorbent dosage on the adsorption of MB (*C*_0_ = 5 mg/L, *t* = 60 min, pH = 7).

In order to investigate the optimum dosage of talc required for the adsorption of MB, a series of experiments were carried out varying only the dosage of adsorbent, and the results are displayed in Figure 9. It can be seen that when the dosage of M-Talc and A-Talc was small, the removal increased rapidly with the increasing dosage when the adsorbent dosage was medium, the removal increased slowly, and finally, at high adsorbent dosage, the removal did not change with increasing adsorbent dosage, meaning that the adsorption reached equilibrium. This is due to the higher dosage adsorbent having a larger specific surface area and more adsorption sites which facilitates the adsorption of the dye molecules, thus enhancing the removal of MB from the solution. In addition, the adsorption of modified talc decreased with increasing adsorbent dosage, which may be due to a decrease in the total adsorption surface area of MB as a result of superposition or agglomeration of adsorption sites [47]. In this work, the optimum dosage of modified talc was 4 g/L.

### 3.7. Adsorption Isotherms

The adsorption isotherm represents the distribution of adsorbate molecules between the solid and the liquid phase at a certain temperature when the adsorption process reaches equilibrium. The shape of the adsorption isotherm reflects the interaction between the dye and the adsorbent and is critical in the adsorption system [48]. In this study, the M-Talc and A-Talc adsorption equilibrium data were fitted with two adsorption isotherm models, Langmuir and Freundlich.

The Langmuir model is a monolayer adsorption isothermal derived from kinetic theory and has been applied to various materials by many researchers. The Langmuir model assumes that the adsorbent surface is homogeneous and all adsorption sites have the same energy, that the adsorption process takes place at a specific site at which dye molecules cannot continue adsorption as long as they occupy one site [49]. The linear equation for the Langmuir model is as follows:

(3) $$\frac{C_{e}}{q_{e}}=\frac{C_{e}}{q_{m}}+\frac{1}{K_{L}q_{m}}$$

where *C_e_* (mg/L) is the equilibrium concentration of the dye, *q_e_* (mg/g) is the adsorption capacity of the adsorbent, *q_m_* (mg/g) is the maximum adsorption capacity and *K_L_* (L/mg) is the Langmuir constant.

The Freundlich model is an empirical equation based on experimental data and is widely used for a heterogeneous surface with different adsorption energies [49]. The linear equation of the Freundlich model is as follows:

(4) $$\log q_{e}=\log K_{F}+\frac{1}{n}\log C_{e}$$

where *n* and *K_F_* ((mg/g) (L/mg)^1/*n*^) represent the Freundlich constants related to the adsorption capacity and intensity. The value of *n* is an indicator to evaluate the goodness of the adsorption process. It is generally accepted that, when 0 < 1/*n* < 1, the adsorption process is easy, and when 1/*n* > 1, it is unfavorable [50].

**Figure 10:**
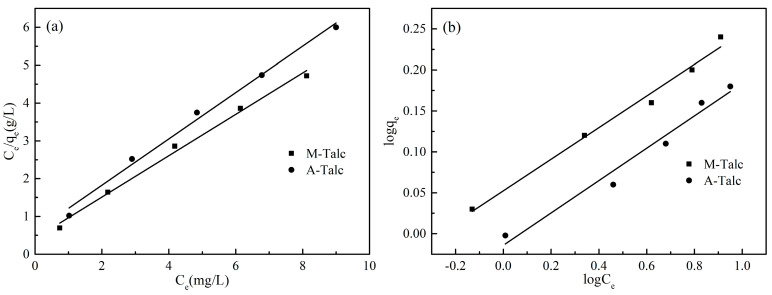
Plots of Langmuir (**a**) and Freundlich (**b**) isotherm models for the adsorption of MB into M-Talc and A-Talc.

The linear plots of the Langmuir and Freundlich isotherms are shown in Figure 10 and the isotherm parameters calculated from the linear equations are given in Table 2. The correlation coefficient values (*R*^2^) fitted by the Langmuir model were better than those fitted by the Freundlich model. Therefore, the Langmuir model was more consistent with the experimental data. The adsorption of MB by the modified talc was monolayer adsorption, and the MB molecules covered the talc surface homogeneously in a single layer, indicating that the specific surface area of the powder adsorbent was proportional to the adsorption capacity. Both the acid-washing modification and ball-milling modification could effectively increase the specific surface area of talc and made its surface adsorption sites more homogeneously distributed. In addition, 1/*n* in the Freundlich model were all less than 1, indicating that the adsorption of MB by the modified talc was easily carried out. Combined with the correlation coefficient values, Freundlich models also provided a favorable fit for the adsorption process.

### 3.8. Adsorption Kinetics

Adsorption kinetics determines the rate of MB removal from aqueous solutions by studying the relationship between contact time and the adsorption capacity of the adsorbent. In this experiment, three kinetic models, pseudo-first-order, pseudo-second-order, and intraparticle diffusion were fitted to investigate the adsorption process and its mechanism.

**Figure 11:**
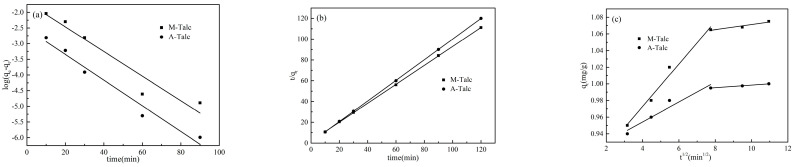
Pseudo-first-order (**a**), pseudo-second-order (**b**), and intraparticle diffusion (**c**) model curves for the adsorption of MB on M-Talc and A-Talc.

The linear equation of the pseudo-first-order model is as follows [51]:

(5) $$\log\left(q_{e}-q_{t}\right)=\log q_{e}-\frac{k_{1}t}{2.303}$$

where *q_e_* (mg/g) and *q_t_* (mg/g) are the adsorption capacity at adsorption equilibrium and the adsorption capacity at time *t* (min), respectively, and *k*_1_ (min^−1^) is the pseudo-first-order model rate constant. The values of *k*_1_ were obtained by plotting log(*q_e_* − *q_t_*) against *t* (Figure 11a). The relevant parameters of the pseudo-first-order model are shown in Table 3. The results showed that the correlation coefficient (*R*^2^) of the pseudo-first-order model was low and the calculated *q_e,cal_* values were not consistent with the experimental data. Therefore, the pseudo-first-order model could not simulate the kinetics of the entire adsorption process well.

The linear equation of the pseudo-second-order model is as follows [51]:

(6) $$\frac{t}{q_{t}}=\frac{1}{k_{2}{q_{e}}^{2}}+\frac{t}{q_{e}}$$

where *k*_2_ (g/mg min) is the pseudo-second-order model rate constant. The values of *k*_2_ were obtained by plotting *t/q_t_* against *t* (Figure 11b). The corresponding calculation parameters are listed in Table 3. It can be seen that the curves fitted by the pseudo-second-order model were well linear with high correlation coefficients compared to the pseudo-first-order model. In addition, the *q_e,cal_* values for the modified talc were closer to the experimental data, indicating that the pseudo-second-order model could better describe the adsorption behavior of MB on the modified talc and that the adsorption process was chemisorption [52].

Since the two kinetic models mentioned above were not sufficient to explain the adsorption mechanism, the experimental results were also fitted using the intraparticle diffusion model summarized by Weber and Morris to investigate the effect of the rate-limiting step of adsorption on the kinetics. The linear equation of the model is as follows [51]:

(7) $$q_{t}=k_{p}t^{1/2}+C$$

where *k_p_* (mg/g min^1/2^) is the diffusion rate constant and *C* is the boundary layer thickness constant. If the curve obtained by plotting *q_t_* against *t*^1/2^ is straight and passes through the origin, i.e., a single linear relationship, it means that intraparticle diffusion is the only rate-limiting step in the adsorption process. Conversely, if the curve is a multilinear plot, the adsorption process is controlled by at least two or more steps. As shown in Figure 11c, the curves fitted by *q_t_* against *t*^1/2^ consist of two line segments, the first segment showing the gradual transfer of MB from solution to the surface of the modified talc, with intraparticle diffusion as the rate-limiting step, while the latter segment indicating that adsorption has reached equilibrium. Neither segment passed through the origin, suggesting that the adsorption process was not entirely controlled by intraparticle diffusion. The mechanism of adsorption of MB by modified talc was complex, and other factors such as boundary layer effects [53] could also affect adsorption.

## 4. Conclusions

In this paper, the surface modification of raw talc was carried out in two different ways, by acid washing and ball milling, and its adsorption performance on the cationic dye MB in aqueous solutions was investigated. A series of characterization results showed that acid activation not only dissolved carbonate impurities in the raw talc but also increased the specific surface area and improved its purity. Ball milling, on the other hand, effectively destroyed the lamellar structure and reduced the particle size of the powder, and CTAB loading on the talc surface played a modifying role. Compared to unmodified talc, both powder modification methods could greatly improve adsorption capacity. The adsorption of MB by modified talc was related to the contact time, pH, dye concentration, and adsorbent dosage. The isotherm fitting based on experimental data indicated that the Langmuir model described the adsorption behavior better and that the adsorption mode was monolayer adsorption. The adsorption kinetic results showed that the pseudo-second-order model was a better fit for the adsorption process. The findings of this work contribute to our understanding of the adsorption process of MB by modified talc and its mechanism. It also demonstrates that modified talc has a promising application in water treatment as a low-cost, effective, and environmentally friendly adsorbent.

## Figures and Tables

**Table 1:** Particle size and specific surface area of raw talc, M-Talc, and A-Talc.

Sample	D_10_ (μm)	D_50_ (μm)	D_90_ (μm)	S_BET_ (m^2^/g)
Raw talc	1.8	13.1	31.6	1.3
M-Talc	0.6	3.1	27.3	3.9
A-Talc	1.5	6.8	29.1	4.5

**Table 2:** The parameters of adsorption isotherms for MB onto M-Talc and A-Talc.

	Langmuir			Freundlich		
Absorbents	*q_m_* (mg/g)	*K_L_* (L/mg)	*R^2^*	*K_F_*	1/*n*	*R* ^2^
M-Talc	1.83	1.32	0.9913	1.13	0.1931	0.9834
A-Talc	1.63	1.03	0.9902	0.97	0.1976	0.9618

**Table 3:** Kinetic parameters for the adsorption of MB onto M-Talc and A-Talc.

		Pseudo-First-Order			Pseudo-Second-Order		
Adsorbents	*q_e,exp_* (mg/g)	*q_e,cal_* (mg/g)	*k*_1_ (min^−1^)	*R* ^2^	*q_e,cal_* (mg/g)	*k*_2_ (g/mg min)	*R* ^2^
M-Talc	1.08	0.19	0.0392	0.9122	1.10	0.4573	0.9999
A-Talc	1.00	0.08	0.0409	0.9629	1.01	1.2623	0.9999

## Data Availability

Not applicable.
